# Testing the Effects of a Preceding Self-Control Task on Decision-Making in Soccer Refereeing

**DOI:** 10.3389/fnins.2021.638652

**Published:** 2021-03-16

**Authors:** Chris Englert, Anna Dziuba, Geoffrey Schweizer

**Affiliations:** ^1^Institute of Education, Department of Educational Psychology, University of Bern, Bern, Switzerland; ^2^Institute of Sports and Sports Science, Karlsruhe Institute of Technology, Karlsruhe, Germany; ^3^Institute of Sports Sciences, Department of Sports Psychology, Heidelberg University, Heidelberg, Germany

**Keywords:** decision-making, ego depletion, self-control, cognitive fatigue, sports, effort, Stroop, refereeing

## Abstract

The present study tested the assumption that the momentary level of self-control strength affects the accuracy rates in a sports-related judgment and decision-making task. A total of *N* = 27 participants rated the veracity of 28 video-taped statements of soccer players who were interviewed by a non-visible referee after a critical game-related situation. In half of the videos, the players were lying, and in the other half, they were telling the truth. Participants were tested twice: once with temporarily depleted self-control strength and once with temporarily available self-control strength (order counterbalanced; measurements separated by exactly 7 days). Self-control strength was experimentally manipulated with the Stroop task. In line with two-process models of information processing, we hypothesized that under ego depletion, information is processed in a rather heuristic manner, leading to lower accuracy rates. Contrary to our expectations, the level of temporarily available self-control strength did not have an effect on accuracy rates. Limitations and implications for future research endeavors are discussed.

## Introduction

Deception in sports is a critical issue as it might decisively change the outcome of a match (Güldenpenning et al., 2017). According to Hsu (1997), deception means “making someone believe something that is not true in order to get what you want” (p. 167). For instance, a wrongfully granted penalty kick during overtime in a tied soccer match will likely determine which team wins the game (Sabag et al., 2018). In sports, lying to the referee can be considered a special form of deception. While research on deception has a long tradition in sports (for an overview see Güldenpenning et al., 2017), and the ability to detect deceit and, especially, lies has been center stage in the criminal justice system (e.g., Akehurst et al., 1996) as well as in educational settings (e.g., Marksteiner et al., 2013) for many years, only recently has the topic of lie detection been addressed in sports-related contexts. This seems rather surprising, given the high potential impact of “successfully” lying to a referee.

Given the impending influence of deceit on the results of a sporting competition, it seems highly important that a referee’s judgment and decision-making take place as accurately as possible. However, as far as we know, there have been very few systematic, experimental studies on referee accuracy rates regarding deception (e.g., Morris and Lewis, 2010; Renden et al., 2014; Aragão e Pina et al., 2018), as most studies on deception in sports have been correlational and, for instance, asked their participants how they would possibly behave in a certain hypothetical situation (e.g., Kavussanu and Ntoumanis, 2003). A notable exception is a study series by Morris and Lewis (2010), in which they first generated a sequence of video clips in which soccer players were instructed to overstate the effects of a tackle by an opposing player. In a subsequent study, neutral observers rated each video clip whether the respective video-taped player had actually been fouled or not. The results revealed that the neutral observers judged the video-clips very accurately. Another experimental study on lie detection was conducted by Englert and Schweizer (2020). Taking a similar approach, the authors first created 28 video clips in which soccer players were either telling the truth or lying regarding two simulated critical game situations. The veracity of each of the 28 video clips was later rated by neutral observers in a series of three studies. The results were rather mixed, as the statements of some of the interviewed players were rather easy to classify, while other players were fairly good at lying. When looking at the accuracy rates of correctly classifying truths and lies in other domains (e.g., the criminal justice system), recent meta-analyses indicate that, overall, individuals are not very accurate at detecting lies, or more precisely, they are only slightly better than the chance level (i.e., accuracy rate of 54%) (e.g., Bond and DePaulo, 2006).

It remains largely unknown which factors influence the accuracy rates of referees. Previous meta-analyses found no empirical evidence that gender, age, expertise, or certain personality traits significantly impacted the accuracy rates (e.g., Aamodt and Custer, 2006; Bond and DePaulo, 2006). In order to identify potential factors, we must first take a closer look at the actual judgment and decision-making process. Dual-process models of information processing assume that there are two different types of information processing when making a judgment (e.g., Chaiken and Maheswaran, 1994; Chaiken and Trope, 1999; Petty et al., 2005) (for an application of dual-process theorizing to the domain of sports see Furley et al., 2015): *Heuristically* (also called *peripheral route*) or *systematically* (also called *central route*). When processing information and making a judgment in a heuristic manner, individuals focus less carefully on the content of a statement and more so on peripheral cues, such as the likability or trustworthiness of the source or simply the number of arguments presented by the source (Petty et al., 2005). On the contrary, systematic information processing allows a person to carefully pay attention and evaluate the quality of the arguments presented (e.g., Chaiken and Trope, 1999). The importance of dual-process models has also been shown in other sport- and exercise-related settings (Furley et al., 2015): for instance, a physically inactive person might have the intention to work out in the evening, but has a negative attitude toward physical exercise and tends to avoid straining physical activities (e.g., Bluemke et al., 2010). In the evening, his/her favorite TV program is on and the person has to make a decision on whether to exercise or not. When making the decision heuristically, the person is less likely to exercise as he/she pays less attention to the positive aspects of physical activity. However, when making the decision systematically, he/she weighs the positive and negative aspects of exercising against one another and is more likely to work out (see also Englert and Rummel, 2016). Taken together, heuristic information processing is less reflective and requires less effort than systematic information processing (Petty et al., 2009; Petty et al., 2005). Previous research from the criminal justice system has reliably shown that judgments are more accurate when taking the systematic information processing route (e.g., Feeley and DeTurck, 1995; Masip et al., 2009; Vrij et al., 2010). This leads to the question: Which factors determine which type of information processing dominates in a given situation? One potential candidate is the level of temporarily available self-control strength, which we will describe in more detail in the following sections (e.g., Davis and Leo, 2012).

According to the strength model, all self-control acts are based on a global metaphorical resource with a limited capacity (e.g., Baumeister et al., 1998; see also Audiffren and André, 2015; André et al., 2019). In this context, self-control means inhibiting certain impulses or response tendencies in order to keep striving for desirable outcomes and to perform at the highest possible level (e.g., Englert, 2017, 2019). Self-control acts include, amongst others, emotion regulation, attention regulation, and most importantly for the present investigation, judgment, and decision-making (Hagger et al., 2010; Samuel et al., 2018) (for an overview, see also Englert, 2017, 2019). It is assumed that after individuals have worked on a self-control task their self-control resources become temporarily depleted for a certain amount of time. During this so-called state of *ego depletion*, following self-control tasks are executed less efficiently as less cognitive effort is likely to be invested (e.g., Baumeister et al., 1998). Given that self-control strength needs to be exerted in order to process information via the cognitively demanding systematic route, previous empirical research has shown that ego depleted individuals tend to process information in a heuristic manner (e.g., Wheeler et al., 2007; Baumeister et al., 2008; Unger and Stahlberg, 2011). In two studies, Reinhard et al. (2013) manipulated ego depletion and found out that ego-depleted participants were more likely to process information heuristically and displayed lower lie detection accuracy rates than non-depleted participants (for similar findings, see also Wheeler et al., 2007; Davis and Leo, 2012).

Based on these empirical findings and theoretical assumptions, we assumed that individuals are more likely to process information heuristically if they had been working on a straining self-control task before (i.e., under ego depletion). As systematic information processing is associated with higher accuracy rates during judgment and decision-making, we tested the hypothesis that depleted individuals are less accurate in correctly classifying ambiguous situations during a soccer match than non-depleted participants (see also Reinhard et al., 2013). In order to test these assumptions, we adopted Englert and Schweizer’s (2020) approach and asked participants at two separate times of measurement to rate the truth of a series of 28 video-taped statements of soccer players, in which they either lied to a referee or told him the truth. At one time of measurement, participants’ self-control strength was experimentally depleted, while it remained intact at the other time of measurement (order counterbalanced).

## Materials and Methods

### Participants

A G^∗^Power (Faul et al., 2007) analysis showed that a sample of *N* = 27 was necessary for detecting at least a medium effect (parameters: *f* = 0.30, α = 0.05, 1−β = 0.85, *r*_repeated measures_ = 0.50, ε = 1). Based on this estimate, a total of *N* = 27 university students from a German university volunteered to partake in the present investigation (16 females, 11 males; *M*_*Age*_ = 27.74 years, *SD*_*Age*_ = 7.17). Three participants had soccer refereeing experience (*M* = 3.67 years, *SD* = 3.79). The study was approved by the local ethics committee, and all participants delivered written informed consent.

### Design, Procedure, and Measures

The participants were tested at two times of measurement exactly 7 days apart under standardized conditions in single sessions on a regular computer in a university lab room. All instructions, video clips, and questionnaires were delivered via an online survey program (Unipark). Each participant was wearing regular stereo headphones, and the sound was played at a constant volume. At one time of measurement, participants’ self-control strength was experimentally depleted (depletion condition), while it remained intact at the other time of measurement (control condition; order counterbalanced). First, participants reported demographic information (i.e., age, sex, and refereeing experience).

Then, self-control strength was experimentally manipulated using the Stroop test, which has been frequently applied in self-control research (e.g., Bray et al., 2012; Englert and Bertrams, 2014). The Stroop test consists of color words which are displayed either in the same font color as the color word (congruent Stroop trial; e.g., the word “red” written in red font color) or in a different font color (incongruent Stroop trial; e.g., the word “red” written in yellow font color); participants need to always name the font color instead of the written color word. It has been reliably shown that in order to ignore the color word and to read the font color instead, self-control needs to be invested, which is why this task has been regularly applied to manipulate self-control strength. In the present study, at both times of measurement, participants first performed a series of 32 practice trials and then worked on 300 incongruent Stroop trials in the depletion condition and on 300 congruent Stroop trials in the control condition. The number of falsely identified Stroop trials and the average response latencies were measured as manipulation checks, assuming that in the depletion condition, participants would make more mistakes and would need longer to answer each trial (in milliseconds) (e.g., Bray et al., 2012; see also Pageaux et al., 2014).

**FIGURE 1:**
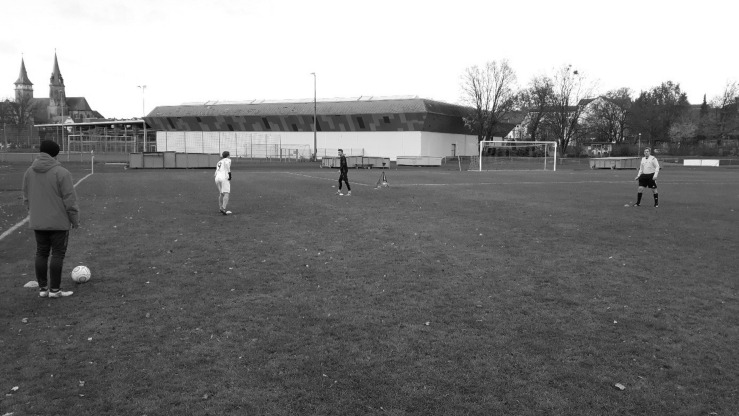
Illustration of the experimental setup for the generation of the stimulus material. The player wearing the jacket is a confederate acting as an attacking player, the player wearing the white jersey is a confederate acting as the teammate of the attacking player, and the player wearing the black jersey is the target player acting as the defender. The referee is standing on the right, observing the scene.

At both times of measurement, following the Stroop task, the participants were informed that they would be watching a series of video clips. These video clips were taken from Englert and Schweizer’s (2020) study, in which the authors created 28 video clips in which male soccer players from a club from the sixth highest league in Germany (out of 11 leagues) were either telling the truth or lying regarding two simulated critical game situations. These simulated game situations took place immediately before an interview with a professional soccer referee (see Figure 1). In both situations, the player acted as a defender as another player played a long pass toward the goal line for his teammate. Once, the defender was asked to not allow the other player to get to the ball and to instead let the ball cross the goal line, which would lead to a goal kick for his team. In the other situation, the instructions were similar with the only difference being that the defender did actually touch the ball last before it passed the goal line. In this latter case, the correct decision would have been a corner kick. However, in both situations, the defender was asked to tell the referee, who had not seen the critical situation, in the subsequent video interview, that the offensive player had touched the ball last and the correct decision was supposedly a goal kick, meaning that the defender was telling the truth in one interview and was lying in the other. The referee asked each player exactly the same questions and was not seen in the video. The participants in the current study did not watch the critical situation, but only the subsequent interview. The participants were also told that each player was in a similar critical situation twice during the same game and would thus be interviewed by the same referee at two separate times. However, the participants were not made aware of the fact that each player was lying in one interview and speaking the truth in the other interview, leading to a total of 14 true statements and 14 lies. On average, each video clip lasted roughly 28 seconds (*M* = 27.5, *SD* = 6.27), and the player’s upper torso, face, and legs could be seen in each clip. The sound quality was the same in all video clips. Participants were further instructed that they would have to rate the veracity of each interview on a continuous scale ranging from 1 (*not at all true*) to 10 (*totally true*) immediately following each video clip (for this procedure, see also Marksteiner et al., 2013). The video clips were displayed in a randomized order immediately after finishing the Stroop task in both conditions. In total, participants rated the veracity of 28 video statements while being ego depleted and the veracity of the same 28 video statements with fully available self-control strength. In order to reduce the likelihood of a learning effect, the two times of measurement were separated by exactly 7 days, and the order of the video presentation was randomized.

Finally, after the second time of measurement, the participants were debriefed and thanked for their participation.

### Data Analysis

Data were analyzed using SPSS (version 27; SPSS Inc., Chicago, IL, United States). We ran paired samples *t*-tests to investigate the assumptions that the depletion condition would perform worse in the Stroop task (i.e., longer response latencies in milliseconds; higher number of Stroop errors) and would be less adept in correctly distinguishing between true and false statements than the control condition. All effect sizes were calculated as Cohen’s *d* (i.e., small effect: *d* = 0.2; medium effect: *d* = 0.5; large effect: *d* = 0.8; Cohen, 1988). For all analyses, statistical significance was accepted as *p* < 0.05.

## Results

### Preliminary Analyses

As expected, the Stroop response latencies in the depletion condition (*M* = 839.08 ms, *SD* = 179.68) were significantly longer than in the control condition (*M* = 717.55 ms, *SD* = 156.80), *t*(26) = 7.02, *p* < 0.0001, *d* = 1.35. Additionally, there was the expected tendency in the number of Stroop errors between the depletion condition (*M* = 7.96, *SD* = 6.00) and the control condition (*M* = 6.59, *SD* = 5.80), which however failed to reach statistical significance, *t*(26) = 1.86, *p* = 0.075, *d* = 0.36. On average, the depletion condition (*M* = 331633.70 ms, *SD* = 69442.08) needed significantly longer to finish the 300 Stroop trials than the control condition (*M* = 295259.67 ms, *SD* = 53785.51), *t*(26) = 3.89, *p* < 0.0001, *d* = 0.75.

### Primary Analyses

In line with Englert and Schweizer’s (2020) approach, for both conditions, we first compared the veracity ratings of the true statements to the veracity ratings of the lies in order to investigate the question of whether participants in both conditions were able to distinguish (on average) between true and false statements (for descriptive statistics see Table 1). In both groups, false statements were rated significantly lower than true statements, indicating that participants in both conditions were able to distinguish between true and false statements (control: *t*(26) = 2.15, *p* = 0.041, *d* = 0.41; depletion: *t*(26) = 4.34, *p* < 0.001, *d* = 0.83).

**TABLE 1:** Mean veracity ratings for the true and false statements, separated by condition (depletion vs. control).

Statement	Depletion condition		Control condition	
		
	*M*	*SD*	*M*	*SD*
True	5.99	0.81	5.83	0.98
False	5.39	0.73	5.32	0.98

**N* = 27. Each video was rated on a continuous scale ranging from 1 (*not at all true*) to 10 (*totally true*).*

Next, in order to investigate potential differences between the depletion and the control conditions, we compared the ratings of the true statements between the two times of measurement (control vs. depletion). Contrary to our hypothesis, the veracity ratings did not differ statistically significantly between the depletion condition (*M* = 5.99, *SD* = 0.81) and the control condition (*M* = 5.83, *SD* = 0.98), *t*(26) = 0.81, *p* = 0.426, *d* = 0.16. There were also no significant differences between the depletion condition (*M* = 5.39, *SD* = 0.73) and the control condition (*M* = 5.32, *SD* = 0.98) in the veracity ratings of the false statements, *t*(26) = 0.39, *p* = 0.703, *d* = 0.07 (see also Table 1).

### Complementary Bayesian Hypothesis Testing

We ran additional Bayesian paired samples *t*-tests, to further investigate whether the differences in the veracity ratings of true and false statements between the depletion and the control condition do not exist (i.e., that the null hypotheses are more likely to be true; for this approach, see also Dienes, 2014; Wagenmakers et al., 2018a,b). For the true statements, a two-sided analysis revealed a Bayes factor (BF01) suggesting that the data were 3.64 times more likely under the null (i.e., the two conditions do not differ in their veracity statements of the true statements) than the alternative hypothesis (i.e., the two conditions differ) with a median effect size of 0.14, which indicates moderate evidence in favor of the null hypothesis. For the false statements, the results indicate that the observed data are 4.58 times more likely under the null (i.e., the two conditions do not differ in their veracity statements of the false statements) than the alternative hypothesis (i.e., the two conditions differ) with a median effect size of 0.07, which indicates moderate evidence in favor of the null hypothesis.

## Discussion

In the present study, we tested the assumption that individuals would be less adept in correctly identifying the veracity of a player’s statement following a critical game situation during a soccer match if they had been working on a straining self-control task beforehand. For that reason, participants rated a series of video statements at two times of measurement, once with fully available self-control strength and once in a state of ego depletion (order counterbalanced). According to two-process models, there are two types of information processing, namely a heuristic and a systematic mode. When judging the veracity of a statement in a heuristic manner, individuals tend to focus on rather invalid cues to deception (e.g., number of statements), while a systematic mode is related to an increased focus on valid cues (e.g., actual content of the statement) and a higher likelihood of classifying a statement correctly (DePaulo et al., 2003; Forrest et al., 2004). But, systematic information processing is effortful and, according to several authors, requires self-control strength (e.g., Wheeler et al., 2007; Baumeister et al., 2008; Unger and Stahlberg, 2011; Davis and Leo, 2012; Reinhard et al., 2013). If one’s self-control resources had been taxed in a previous task, he/she is less likely to have the necessary self-control strength to process information systematically and will tend to process heuristically instead. However, the results did not support our hypothesis as there were no statistically significant differences in the accuracy rates between the control and the depletion condition.

When investigating why the control and the depletion condition did not differ regarding their veracity ratings, it is important to emphasize that in both conditions, participants actually could differentiate between true and false statements (although not very strongly). This can be considered a necessary prerequisite for testing our main hypothesis: If participants in the control condition cannot distinguish between true and false statements, then they cannot get worse in the depletion condition. Given that this prerequisite was met, how can we then explain that participants in the depletion and the control conditions did not differ, considering that the study was adequately powered and that the depletion manipulation was effective? One potential explanation for this pattern is that participants in the control condition did rely on heuristic processing as well. This would both explain why participants were not able to distinguish more strongly between false and true statements (because doing so would require more systematic processing) and why they did not get worse in the depletion condition. To address this issue, further research might want to employ not only a condition that is supposed to decrease systematic and to increase heuristic processing (such as the depletion condition in the present research), but furthermore a condition that is supposed to increase systematic processing. This might be accomplished by incentivizing participants, for example (see also Beckmann, 2020).

Another potential explanation might be the low level of expertise/experience of the participants in our study (only three participants had soccer refereeing experience), as one might reason that participants with soccer refereeing experience are better at correctly judging player statements (e.g., MacMahon et al., 2007; Moore et al., 2019). Even though several large-scale studies from the criminal justice system and educational psychology have reliably demonstrated that the raters’ expertise does not affect their accuracy rates (e.g., Aamodt and Custer, 2006; Bond and DePaulo, 2006), future studies should investigate whether the same is true in sports-related judgment and decision-making situations.

We would also like to address the fact that the depletion condition took significantly longer to finish the Stroop task than the control condition. This matter seems especially important, as a recent study by Boat et al. (2020) revealed that longer Stroop task durations were related to lower performance in a subsequent self-control task. However, in the current study we did not find an effect of the different Stroop task durations on the veracity ratings. Future studies should continue to dig deeper into the effects of different self-control task durations on performance (see also Wolff et al., 2021).

Individuals do not only differ in their levels of temporarily available self-control, but also in their general self-control abilities, meaning that some are simply better at regulating themselves than others (i.e., trait self-control; Tangney et al., 2004). In general, individuals with higher levels of trait self-control are more adept at volitionally controlling their impulses and focusing on the task at hand (e.g., De Ridder et al., 2012). In the current study, we did not measure trait self-control strength; however, given the fact that we applied a repeated measures design, we assume that trait self-control strength did not play a major part in our study. It has to be noted that the validity of the ego depletion effect itself has been questioned on theoretical and empirical grounds. On an empirical level, some recent large-scale replication studies did not find reliable statistical evidence for the ego depletion effect (e.g., Hagger et al., 2016; Blázquez et al., 2017). For instance, Vohs et al. (2021) conducted a preregistered replication report with over 3,500 participants from 36 labs worldwide. While participants with depleted self-control did not differ significantly from the non-depleted participants in terms of their performance, depleted participants did feel more fatigued than control participants. So, why did depleted participants feel fatigued while their actual performance did not suffer from their depletion? It might be reasonable to assume that the dependent variable in the Vohs et al.’s study (Cognitive Estimation Test) (Bullard et al., 2004) was not self-control demanding *enough*. If the dependent measure only requires minimal effort, it is highly unlikely to be affected by a straining preceding self-control task (see also Loschelder and Friese, 2016). In a similar fashion, in our study rating the videos systematically might not place sufficiently high self-control demands on one’s self-control resources, thus making it more difficult to find statistically significant differences between the depleted and the non-depleted conditions. Furthermore, while the results of the Stroop test revealed the expected differences between the depletion and the control condition, we did not apply an additional manipulation check measuring the level of perceived depletion following the Stroop task. This notion seems especially important, as for instance Clarkson et al. (2010) have demonstrated that participants who perceived themselves as being more depleted performed worse in following self-control acts than participants who perceived themselves as being less depleted (see also Wright and Mlynski, 2019). Even though previous studies have reliably shown that participants reported significantly higher levels of perceived depletion after the incongruent Stroop task compared to the congruent one (e.g., Hagger et al., 2010), future studies should apply additional manipulation checks to test the effectiveness of the respective ego depletion manipulation.

On a theoretical level, several researchers argue that the assumption of a limited metaphorical self-control resource is not appropriate and cannot be adequately tested empirically (for a discussion, see also Eronen and Bringmann, 2021). For instance, the process model by Inzlicht and Schmeichel (2012, 2016) postulates that a primary self-control act does not deplete limited resources but rather instigates shifts in motivation (i.e., the person does not want to work on another straining task), emotions (i.e., the person perceives other straining tasks as rather negative), and attention (i.e., impaired attention regulation), which ultimately affects performance in subsequent self-control tasks. In a similar fashion, according to the behavioral restraint extension of the general fatigue analysis (e.g., Wright and Agtarap, 2015; Wright and Mlynski, 2019), the amount of self-control (i.e., restraint intensity) one can or, more precisely, is willing to invest in a given task is not dependent on temporarily available self-control resources. Rather, it is a function of perceived fatigue, task difficulty (i.e., the magnitude of an unwanted urge), and success importance (i.e., the importance of resisting the urge), with associated cardiovascular responses following (i.e., changes in systolic and diastolic blood pressure as well as mean arterial pressure; Wright et al., 2012). Therefore, fatigue does not automatically lead to less effort or impaired self-control performance (e.g., Wright et al., 2013). For instance, if a fatigued person thinks that success in an upcoming task is highly unlikely and that success is not especially important, he or she is unlikely to invest high amounts of effort which will eventually lead to impaired performance. However, if the same person views success in the upcoming task as likely and important, he or she will be willing to invest more effort and perform at a higher level. Assessing these additional psychological and physiological parameters specified in the process model as well as the behavioral restraint extension of the general fatigue analysis might shed some light on the actual mechanisms contributing to our present pattern of results.

Taken together, even though we did not find statistically significant differences between the control and the depletion condition in accuracy rates, we do consider the present findings to be highly informative. First, they suggest that participants are not necessarily worse at detecting lies in sports when in a state of ego-depletion. Second, the present findings suggest fruitful avenues for further research (e.g., different manipulations for systematic and heuristic processing). Third, it adds to the recent discussion surrounding the ego depletion effect, indicating that systematic information processing might be less prone to be affected by states of ego depletion. Fourth, it highlights the necessity to dig deeper into the psychological and physiological mechanisms potentially affecting self-control performance.

## Data Availability Statement

The raw data supporting the conclusions of this article will be made available by the authors, without undue reservation.

## Ethics Statement

The studies involving human participants were reviewed and approved by the Ethics Board of the University of Bern. The patients/participants provided their written informed consent to participate in this study. Written informed consent was obtained from the individual(s) for the publication of any potentially identifiable images or data included in this article.

## Author Contributions

CE, AD, and GS equally contributed to the conceptualization of the study and review of relevant related work. CE, GS, and AD analyzed and interpreted the data. CE and GS prepared the draft manuscript. AD provided the critical revisions. All authors approved the final version of the manuscript and agreed with the order of presentation of the authors.

## Conflict of Interest

The authors declare that the research was conducted in the absence of any commercial or financial relationships that could be construed as a potential conflict of interest.
